# Supplementary material for: The intersection of the retrieval state and internal attention
Source: Nat Commun. 2023 Jun 29;14:3861. doi: 10.1038/s41467-023-39609-9 (PMC10310828; doi:10.1038/s41467-023-39609-9)
Supplement: Supplementary file 1 — Supplemental Information [file 41467_2023_39609_MOESM1_ESM.pdf]

**The intersection of the retrieval state and internal attention.**

Supplementary Information.

Nicole M. Long[1], Ph.D.

1: Department of Psychology, University of Virginia 22904

Here all mnemonic-state based analyses reported in the main manuscript were recalculated utilizing the full training (N=100) set of participants from the mnemonic state task. Retrieval state evidence is modulated across the stimulus interval (Figure 1A) and during the delay interval separately for cued and neutral trials (Figure 1B-D; Table 1). Retrieval evidence is correlated with cue direction evidence (Figure 1E). Retrieval evidence is modulated during the response interval (Figure 2A; Table 2), correlated with probe information (Figure 2B), and predicts target detection reaction times (Figure 2C).

**Table 1. Delay interval retrieval state evidence as a function of cue type, SOA, and time, repeated measures ANOVAs.**

| | SOA = 200 | | | | SOA = 400 | | | | SOA = 800 | | | |
|---|---|---|---|---|---|---|---|---|---|---|---|---|
| Effect | df | *F* | *p* | ${\eta_p}^2$ | df | *F* | *p* | ${\eta_p}^2$ | df | *F* | *p* | ${\eta_p}^2$ |
| Main effect of time | 1,36 | 41.76 | <0.001 | 0.54 | 3,108 | 24.21 | <0.001 | 0.40 | 7,252 | 49.52 | <0.001 | 0.58 |
| Main effect of cue | 1,36 | 0.534 | 0.469 | 0.01 | 1,36 | 2.167 | 0.15 | 0.06 | 1,36 | 5.744 | 0.022 | 0.14 |
| Interaction of time × cue | 1,36 | 0.008 | 0.927 | 0.0002 | 3,108 | 0.054 | 0.983 | 0.001 | 7,252 | 3.487 | 0.001 | 0.09 |

**Table 2. Response interval retrieval state evidence as a function of cue type, SOA, and time, repeated measures ANOVAs.**

| | | Retrieval Evidence | | |
|---|---|---|---|---|
| Effect | df | *F* | *p* | ${\eta_p}^2$ |
| Main effect of time | 4,144 | 86.16 | < 0.001 | 0.71 |
| Main effect of cue | 2,72 | 1.681 | 0.193 | 0.04 |
| Main effect of SOA | 2,72 | 25.72 | < 0.001 | 0.42 |
| Interaction of time × cue | 8,288 | 1.576 | 0.131 | 0.04 |
| Interaction of time × SOA | 8,288 | 11.3 | < 0.001 | 0.24 |
| Interaction of cue × SOA | 4,144 | 3.481 | 0.01 | 0.09 |
| Interaction of cue × SOA × time | 16,576 | 0.344 | 0.992 | 0.009 |

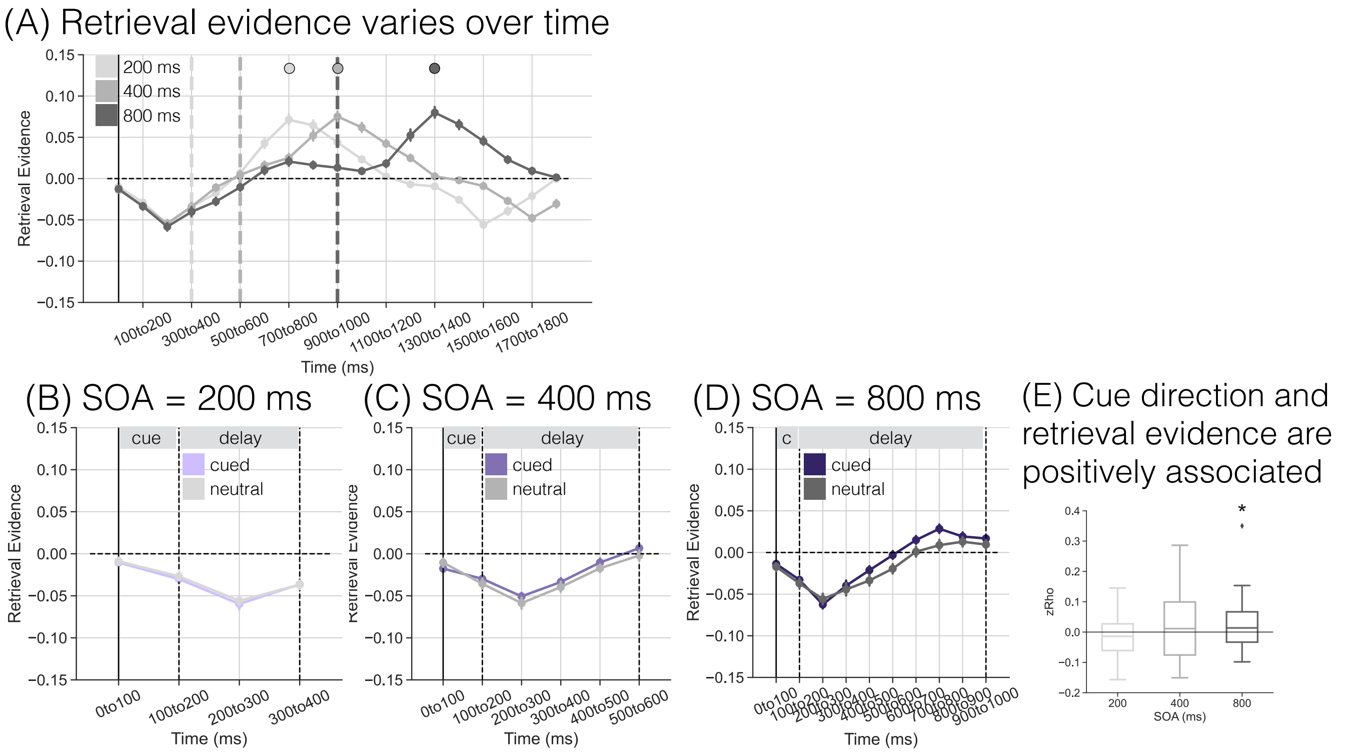


Figure 1. **Delay Interval Retrieval State Evidence.** The mnemonic state classifier was trained utilizing the full set of n=100 participants who completed the mnemonic state task. Classifier was tested on the attention task data (n=37 participants). Positive y-axis values indicate greater retrieval state evidence. The solid vertical line at time 0 to 100 ms indicates the onset of the cue. The vertical dashed lines indicate the onset of the probe, which varies as a function of stimulus onset asynchrony (SOA). **(A)** All trials are included; data have been averaged over cue type. Note that trial duration varies as a function of SOA, meaning that shorter SOA trials (200 and 400 ms) will end prior to the final time window shown. Across all SOAs, the trial initially begins with a decrease in retrieval that persists for approximately 500 ms, followed by an increase in retrieval that is maximal around the time point when the average response is made (indicated by the circles). **(B-D)** Each panel shows retrieval evidence separated by cue type (purple: cued, average of valid/invalid; grey: neutral) across the 100 ms cue and variable delay intervals. **(B)** There is no difference in retrieval state evidence between cued and neutral trials for the 200 ms SOA condition. **(C)** There is no difference in retrieval state evidence between cued and neutral trials for the 400 ms SOA condition. **(D)** Retrieval evidence is greater for cued compared to neural trials for the 800 ms SOA condition. **(E)** I performed trial level Pearson correlations between cue direction evidence (left or right with neutral evidence as a baseline, see Methods) and retrieval state evidence. There was a significant correlation between cue direction evidence and retrieval state evidence for the 800 ms SOA condition (two tailed, paired *t*-test, $p = 0.018$). Error bars represent standard error of the mean. Box-and-whisker plots show median (center line), upper and lower quartiles (box limits), 1.5x interquartile range (whiskers) and outliers (diamonds). Two tailed, paired *t*-test, * $p < 0.05$, uncorrected.

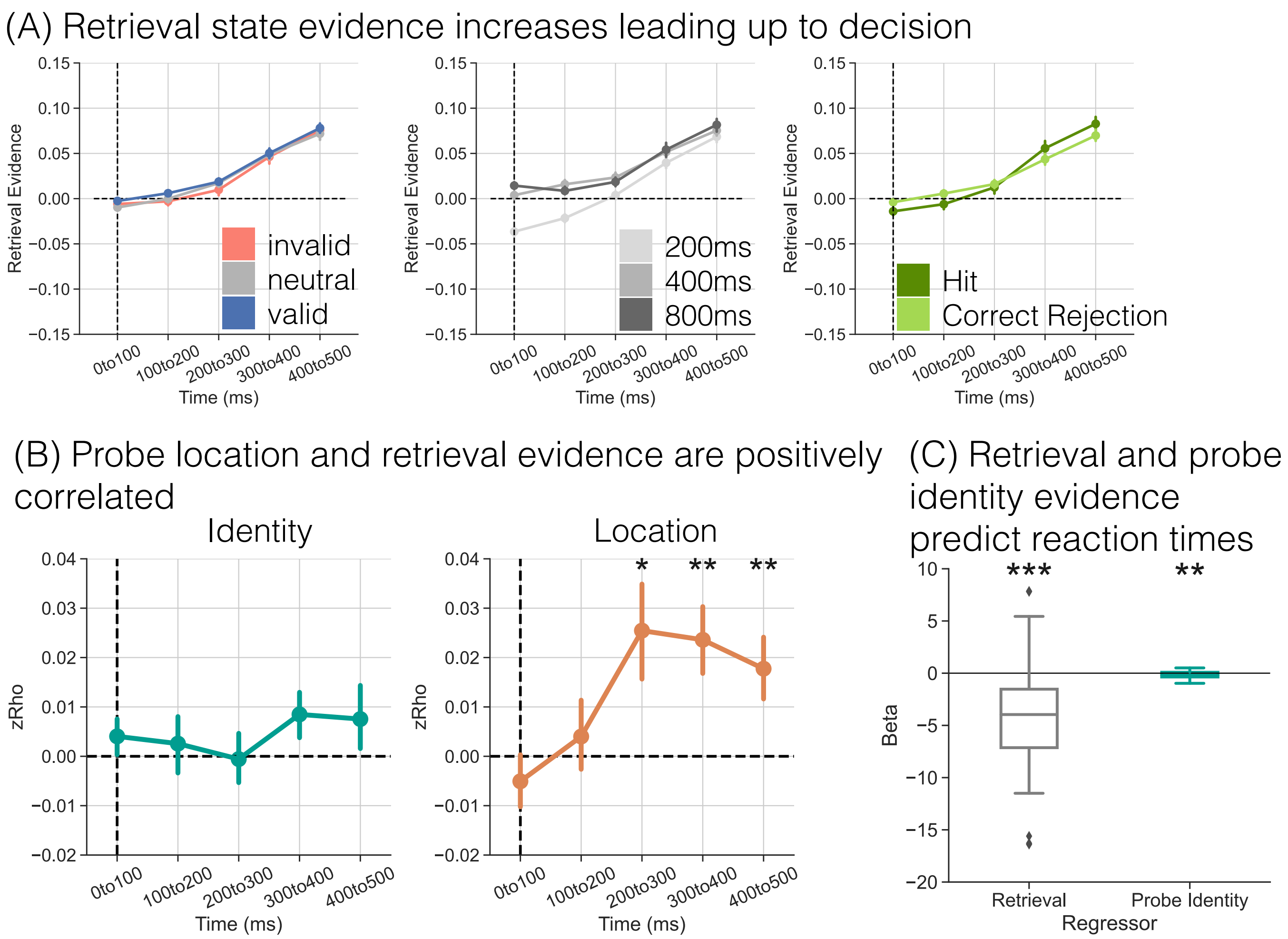


**Figure 2. Response Interval Retrieval State Evidence.** The mnemonic state classifier was trained utilizing the full set of n=100 participants who completed the mnemonic state task and tested on the attention task data (n=37 participants). **(A)** Each panel shows probe-locked retrieval state evidence; positive values indicate greater retrieval state evidence. The dashed vertical line at time 0 to 100 ms indicates the onset of the probe (cross target or plus lure). The left panel shows retrieval state evidence separated by cue type (invalid, red; neutral, grey; valid, blue). The middle panel shows retrieval state evidence separated by SOA (200, 400, 800ms). The right panel shows retrieval state evidence separately for hits (cross targets to which participants responded; dark green) and correct rejections (plus lures to which participants withheld a response; light green). **(B)** I performed trial level Pearson correlations between probe identity (cross, plus) evidence (left panel) or probe location (left, right) evidence (right panel) and retrieval state evidence across the response interval. There was a significant positive correlation between probe location and retrieval evidence during the 200-500ms of the response interval (two-tailed, paired *t*-tests, 200-300ms: $p = 0.014$, 300-400ms: $p = 0.001$, 400-500ms: $p = 0.009$, FDR corrected). **(C)** I performed multiple linear regression in which we used retrieval evidence and probe identity evidence during the 300-400ms time window to predict reaction times (RTs). Only trials with RTs $>$ 400 ms are included. There were significant negative betas for both regressors (two-tailed, paired *t*-tests, retrieval evidence: $p < 0.001$, probe identity evidence: $p = 0.002$, FDR corrected) meaning that more retrieval evidence and more probe identity evidence predict faster RTs. Error bars represent standard error of the mean. Box-and-whisker plots show median (center line), upper and lower quartiles (box limits), 1.5x interquartile range (whiskers) and outliers (diamonds). Two-tailed, paired *t*-tests * $p < 0.05$; ** $p < 0.01$; *** $p < 0.001$; FDR corrected.
