# Supplementary material for: The intersection of the retrieval state and internal attention
Source: Nat Commun. 2023 Jun 29;14:3861. doi: 10.1038/s41467-023-39609-9 (PMC10310828; doi:10.1038/s41467-023-39609-9)
Supplement: Supplementary file 2 — Reporting Summary [file 41467_2023_39609_MOESM2_ESM.pdf]

nature portfolio

Corresponding author(s): Nicole M. Long

Last updated by author(s): May 6, 2023

Reporting Summary

Nature Portfolio wishes to improve the reproducibility of the work that we publish. This form provides structure for consistency and transparency in reporting. For further information on Nature Portfolio policies, see our Editorial Policies and the Editorial Policy Checklist.

Statistics

For all statistical analyses, confirm that the following items are present in the figure legend, table legend, main text, or Methods section.

n/a	Confirmed	
☐	☒	The exact sample size (n) for each experimental group/condition, given as a discrete number and unit of measurement
☐	☒	A statement on whether measurements were taken from distinct samples or whether the same sample was measured repeatedly
☐	☒	The statistical test(s) used AND whether they are one- or two-sided<br>*Only common tests should be described solely by name; describe more complex techniques in the Methods section.*
☒	☐	A description of all covariates tested
☐	☒	A description of any assumptions or corrections, such as tests of normality and adjustment for multiple comparisons
☐	☒	A full description of the statistical parameters including central tendency (e.g. means) or other basic estimates (e.g. regression coefficient) AND variation (e.g. standard deviation) or associated estimates of uncertainty (e.g. confidence intervals)
☐	☒	For null hypothesis testing, the test statistic (e.g. F, t, r) with confidence intervals, effect sizes, degrees of freedom and P value noted<br>*Give P values as exact values whenever suitable.*
☒	☐	For Bayesian analysis, information on the choice of priors and Markov chain Monte Carlo settings
☒	☐	For hierarchical and complex designs, identification of the appropriate level for tests and full reporting of outcomes
☐	☒	Estimates of effect sizes (e.g. Cohen's d, Pearson's r), indicating how they were calculated

Our web collection on statistics for biologists contains articles on many of the points above.

Software and code

Policy information about availability of computer code

Data collection	Data collection code area combination of open source Python-based codes, including the SMILE package (1.0.0), and custom Python codes. All custom code are publicly available at https://osf.io/du56c/
Data analysis	Data analysis code are a combination of open source Python codes and custom python codes. Data analysis utilized sklearn (0.24.2). All custom code are publicly available at https://osf.io/du56c/

For manuscripts utilizing custom algorithms or software that are central to the research but not yet described in published literature, software must be made available to editors and reviewers. We strongly encourage code deposition in a community repository (e.g. GitHub). See the Nature Portfolio guidelines for submitting code & software for further information.

Data

Policy information about availability of data

All manuscripts must include a data availability statement. This statement should provide the following information, where applicable:

- Accession codes, unique identifiers, or web links for publicly available datasets
- A description of any restrictions on data availability
- For clinical datasets or third party data, please ensure that the statement adheres to our policy

The raw data generated in this study have been deposited in the Open Science Foundation database at https://osf.io/du56c/. Source data are provided with this paper.

Research involving human participants, their data, or biological material

Policy information about studies with human participants or human data. See also policy information about sex, gender (identity/presentation), and sexual orientation and race, ethnicity and racism.

Reporting on sex and gender	We report the gender of human participants. Gender was determined based on self-report. Findings apply to all genders, there are no sex- or gender-based analyses. Of 40 participants, 31 self-reported as female and 9 self-reported as male.
Reporting on race, ethnicity, or other socially relevant groupings	There are no race, ethnicity, or other socially relevant grouping based analyses.
Population characteristics	There are no a priori hypotheses regarding sex/gender, race/ethnicity, and/or age and the observed effects are not expected to differ across any of these dimensions. Thus, there are no analyses performed based on population characteristics
Recruitment	Participants were recruited from the University of Virginia community via flyers. Individuals who choose to participant in research studies may systematically differ from those who do not; however, the neural mechanisms reported in this study are expected to reflect basic biological mechanisms that should not be impacted by potential self-selection bias.
Ethics oversight	The University of Virginia Institutional Review Board for Social and Behavioral Research approved the protocol

Note that full information on the approval of the study protocol must also be provided in the manuscript.

Field-specific reporting

Please select the one below that is the best fit for your research. If you are not sure, read the appropriate sections before making your selection.

☒ Life sciences ☐ Behavioural & social sciences ☐ Ecological, evolutionary & environmental sciences

For a reference copy of the document with all sections, see nature.com/documents/nr-reporting-summary-flat.pdf

Life sciences study design

All studies must disclose on these points even when the disclosure is negative.

Sample size	Sample size was determined a priori based on prior EEG studies that have been conducted (e.g. Long & Kuhl, 2019). The sample size is consistent with contemporary EEG studies of similar design. A post-hoc power analysis on a key finding from the manuscript in which neural signals facilitate behavioral responses achieved a power of 0.99.
Data exclusions	Three participants were excluded from the analyses. One participant was excluded due to data loss. Two participants were excluded due to poor signal quality (impedances were above the threshold of 50 kΩ).
Replication	There was no attempted replication of the reported results, but note that the behavioral findings are consistent with the literature.
Randomization	A within-subjects designed was used, thus there are no experimental groups. All participants experienced all conditions. Condition presentation order was randomized within participant
Blinding	Blinding is not relevant as all participants experienced all conditions.

Reporting for specific materials, systems and methods

We require information from authors about some types of materials, experimental systems and methods used in many studies. Here, indicate whether each material, system or method listed is relevant to your study. If you are not sure if a list item applies to your research, read the appropriate section before selecting a response.

Materials & experimental systems

n/a	Involved in the study	
☒	☐	Antibodies
☒	☐	Eukaryotic cell lines
☒	☐	Palaeontology and archaeology
☒	☐	Animals and other organisms
☒	☐	Clinical data
☒	☐	Dual use research of concern
☒	☐	Plants

Methods

n/a	Involved in the study	
☒	☐	ChIP-seq
☒	☐	Flow cytometry
☒	☐	MRI-based neuroimaging
